# Cybervictimization, self-esteem, and cyberaggression endorsement in Thai university students: evidence from a two-part zero-inflated model

**DOI:** 10.3389/fpsyg.2026.1863842

**Published:** 2026-09-11

**Authors:** Chawisa Suradom, Peerakarn Na Bangsai, Kewalin Wattanatchariya, Mathuramat Seesen, Xu Quan, Nahathai Wongpakaran, Tinakon Wongpakaran

**Affiliations:** 1 Department of Psychiatry, Faculty of Medicine, Chiang Mai University, Chiang Mai, Thailand; 2 Department of Community Medicine, Faculty of Medicine, Chiang Mai University, Chiang Mai, Thailand; 3 Master of Science Program (Mental Health), Multidisciplinary and Interdisciplinary School (MIdS), Chiang Mai University, Chiang Mai, Thailand

**Keywords:** cyberaggression, cybervictimization, self-esteem, university students, zero-inflated model resilience

## Abstract

**Background:**

Cybervictimization and cyberaggression are increasingly recognized as public health concerns among university students; however, evidence from Thailand remains limited.

**Objective:**

This study examined whether cybervictimization was associated with cyberaggression endorsement and whether self-esteem moderated this association. We used a two-part zero-inflated Poisson (ZIP) framework to distinguish between (a) a structural-zero/no-endorsement process and (b) the extent of cyberaggression endorsement among individuals at risk.

**Methods:**

A cross-sectional online survey was conducted in January 2023 among university students in Chiang Mai, Thailand (*N* = 414). Cybervictimization was assessed using the victimization subscale of the Thai version of the Cyberaggression–Perpetration and Victimization Scale. Cyberaggression was operationalized as a bounded behavioral endorsement count (AGGRESSO; range = 0–5), reflecting the number of cyberaggressive perpetration behaviors endorsed during the past month. Self-esteem was assessed using the Thai Rosenberg Self-Esteem Scale. Competing Poisson, negative binomial, and ZIP models were compared using information criteria.

**Results:**

Model comparisons supported the ZIP specification (AIC = 484.143; BIC = 516.350). In the ZIP count component, higher self-esteem was associated with a lower extent of cyberaggression endorsement among at-risk individuals (*b* = −0.078, *p* = 0.005). Cybervictimization (*b* = 0.048, *p* = 0.386) and the cybervictimization × self-esteem interaction (*b* = 0.015, *p* = 0.180) were not statistically significant in this component.In the zero-inflation component, the cybervictimization × self-esteem interaction was statistically significant (*b* = −0.106, *p* = 0.050), while the main effect of cybervictimization was marginal (*b* = −1.406, *p* = 0.054). These findings indicate that the association between cybervictimization and structural non-endorsement—equivalently, the likelihood of any cyberaggression endorsement—differed according to self-esteem. Sensitivity analyses using binary and ordinal outcome specifications produced a consistent pattern: cybervictimization and its interaction with self-esteem were most evident for the involvement aspect of cyberaggression (i.e., any endorsement), rather than the extent of endorsement.

**Conclusion:**

Cybervictimization was more strongly related to cyberaggression involvement than to the extent of cyberaggression endorsement among at-risk individuals, whereas self-esteem showed a component-specific pattern. These findings demonstrate the value of distinguishing between involvement and endorsement extent in cyberaggression research. University prevention efforts may benefit from timely support following cybervictimization, together with interventions that strengthen self-regulation and adaptive coping resources.

## 1 Introduction

Cybervictimization and cyberaggression are increasingly salient forms of online interpersonal harm. Enacted through digital communication, these behaviors include hostile, humiliating, exclusionary, and privacy-violating acts (Willard, 2007) and are widely recognized as social and public health concerns. Cyberaggression is typically defined as intentional harm inflicted through digital means, regardless of repetition or power imbalance (Englander et al., 2017; Corcoran et al., 2015). Cybervictimization, in turn, has been consistently linked to a broad range of psychological, behavioral, and academic difficulties (Kasturiratna et al., 2025; Marciano et al., 2020; Gardella et al., 2017), underscoring its impact on individual wellbeing and functioning.

These concerns are especially relevant among emerging adults, one of the most digitally engaged groups worldwide. Recent estimates indicate that approximately 82% of individuals aged 15–24 use the internet globally (International Telecommunication Union [ITU], 2025). In highly digitalized contexts such as Thailand, widespread internet use is accompanied by substantial exposure to cyberbullying-related experiences among university students (e.g., Wongchinsri et al., 2022; Saengcharoensap and Rujiprak, 2021). Yet despite the relevance of harmful online experiences to university populations—who spend substantial time on social media and peer-based digital communication—empirical research has focused predominantly on children and adolescents (e.g., Lee et al., 2026; Tran et al., 2023; Marciano et al., 2020), leaving university students comparatively understudied. Clarifying risk and protective processes in university samples is important not only for developmental understanding but also for campus prevention and student support services.

A core question in this literature is why cybervictimization is associated with cyberaggression. Longitudinal evidence suggests that cybervictimization predicts subsequent cyberaggression even after accounting for prior aggression (Wright and Li, 2013). Complementary findings from university-based studies also indicate positive associations between cybervictimization and cyberaggression and suggest that reciprocal relations may occur over time (Zhan et al., 2022; Yildiz Durak and Saritepeci, 2020). However, the victim-to-perpetrator pathway is not inevitable: many victimized individuals do not go on to aggress against others online. This variability highlights the need to identify psychological processes and resources that shape whether and how victimization experiences are translated into aggressive responding.

Prior research has identified several intrapersonal mechanisms that may connect cybervictimization to cyberaggression. Cybervictimization has been shown to elicit negative emotional responses such as anger, which can increase the likelihood of aggressive behavior (Ak et al., 2015). Cognitive processes such as anger rumination may further reinforce this pathway over time (Camacho et al., 2021). Stress-related processes and maladaptive coping (e.g., revenge motivations) have also been implicated in the progression from victimization to cyberaggression (Quintana-Orts et al., 2020). In addition, regulatory and moral factors—including emotion regulation and social responsibility—may attenuate or exacerbate aggressive responding following victimization (Uddin and Rahman, 2022; Zhan et al., 2022). Taken together, these findings suggest that the transition from cybervictimization to cyberaggression is contingent on individual psychological processes and resources. However, comparatively less attention has been paid to broader self-evaluative resources that may shape appraisals of victimization and subsequent behavioral responses.

Self-esteem may be particularly important in this regard. Self-esteem—an individual’s global evaluation of self-worth (Orth and Robins, 2013), is closely tied to perceptions of social acceptance and rejection (Leary et al., 1995). In cyberbullying research, lower self-esteem has been more consistently associated with victimization than with perpetration, whereas findings for aggressors have been less uniform (Lei et al., 2020; Patchin and Hinduja, 2010). More broadly, longitudinal evidence indicates that low self-esteem functions not merely as a correlate but also as a vulnerability factor, while adverse experiences may in turn erode self-esteem (Budiarto and Helmi, 2021; Muris and Otgaar, 2023). In the context of cybervictimization, experiences of humiliation, rejection, and denigration may undermine self-esteem by conveying negative messages about one’s social worth (Kim and Lee, 2020). At the same time, perspectives emphasizing ego threat suggest that threats to social value and self-worth may elicit defensive responding; thus, under some conditions, victimization may be linked to stronger aggressive reactions among individuals with more positive self-evaluations. Consistent with this view, emerging longitudinal evidence indicates that self-esteem may shape how victimized individuals respond to such experiences, including whether victimization is later expressed through aggressive behavior (Choi and Park, 2018). Accordingly, self-esteem may moderate the cybervictimization–cyberaggression association. Recent evidence among college students likewise indicates that cybervictimization is associated with cyberbullying perpetration and that self-esteem may be relevant to this association, alongside affective responses such as trait anger (Zhang et al., 2025). In addition, prior research suggests that involvement in cybervictimization and cyberaggression may differ by gender and sexual orientation, and sexual and gender minority students may experience higher rates of peer harassment in online contexts. Therefore, we collected gender and sexual orientation to characterize the sample and to aid interpretation and comparability with previous studies (Abreu and Kenny, 2018).

Methodological differences across studies may also contribute to inconsistent findings. Cyberaggression is not only a question of whether individuals engage in aggression but also the extent of endorsement once involved. Many studies have operationalized cyberaggression as a dichotomous outcome and used logistic regression to model involvement only (e.g., Luzayisu and Tungu, 2023), which can obscure meaningful variation in endorsement levels. At the same time, cyberaggression measures are often behavior-endorsement composites that are bounded and show substantial excess zeros, with many participants reporting no perpetration and a smaller subset reporting multiple behaviors. Such distributions motivate the use of two-part count frameworks that distinguish predictors of any endorsement from predictors of the level of endorsement among those at risk (Kang et al., 2021; Cho et al., 2019). In the present study, cyberaggression is modeled as a bounded endorsement count with a high proportion of zero responses; therefore, we apply a two-part zero-inflated count approach (ZIP) that separates a zero-inflation component (structural zero/no endorsement) from a count component (extent of endorsement among at-risk individuals). Importantly, applying a moderation perspective to this two-part framework allows more precise inferences about whether self-esteem shapes (a) the likelihood of moving away from structural non-endorsement and/or (b) the extent of endorsement among those at risk.

### 1.1 Novelty and contribution

The present study extends existing research in three ways. First, it focuses on Thai university students, an understudied group relative to adolescents despite high digital engagement and relevance for campus prevention efforts. Second, it treats cyberaggression as a bounded, zero-heavy behavioral endorsement count and applies a two-part zero-inflated framework that distinguishes predictors of any endorsement (structural non-endorsement vs. involvement) from predictors of endorsement extent among at-risk individuals. Third, rather than examining self-esteem only as a correlate, the study tests self-esteem as a moderator across both components of the two-part model, clarifying whether self-esteem conditions the cybervictimization–cyberaggression association primarily through involvement, endorsement extent, or both. This component-specific approach is intended to improve theoretical precision and strengthen the practical interpretability of findings for university-based prevention and support.

In light of these theoretical and methodological considerations, the present study examines the association between cybervictimization and cyberaggression among Thai university students and tests self-esteem as a moderator within a two-part zero-inflated framework. Accordingly, we propose the following hypotheses:

*H1*: Cybervictimization will be positively associated with cyberaggression endorsement.

*H2a*: Self-esteem will moderate the association between cybervictimization and the probability of structural non-endorsement such that the negative association between cybervictimization and belonging to the structural-zero/no-endorsement subgroup will be stronger at higher self-esteem.

*H2b*: Self-esteem will moderate the association between cybervictimization and the extent of cyberaggression endorsement among at-risk individuals such that the positive association between cybervictimization and endorsement extent will be stronger at higher self-esteem.

## 2 Materials and methods

### 2.1 Study population and sample

This cross-sectional study was conducted in January 2023 among university students at Chiang Mai University (CMU), Chiang Mai, Thailand. Participants were recruited via online convenience sampling using advertisements posted in CMU student communities and university Facebook groups. This recruitment approach was selected to maximize feasibility and accessibility within the study timeframe and to support anonymous participation when assessing sensitive behaviors (cyberaggression), by allowing students to participate without in-person contact. Accordingly, the study was designed to estimate associations among variables within the recruited CMU sample rather than to generate prevalence estimates generalizable to all Thai university students.

Eligibility criteria were: (a) age ≥ 18 years; (b) current enrollment at CMU; (c) ability to read and communicate in Thai; and (d) provision of electronic informed consent. Participants were excluded if they reported a diagnosis of schizophrenia or current psychotic symptoms (e.g., hallucinations or delusions) for which they were receiving treatment at the time of survey participation. Because recruitment used convenience sampling and the number of individuals who viewed the online invitations could not be determined, we did not set an *a priori* recruitment target; instead, we conducted a sensitivity (power) analysis. Under α = 0.05 (two-tailed), power = 0.90, and Cohen’s *f*^2^ = 0.05 (small-to-moderate) for regression-type moderation models with cybervictimization, self-esteem, and their interaction, the required sample size was approximately N ≈ 263. The achieved analytic sample (*N* = 414) therefore provided adequate power for the planned analyses. A total of 414 participants were included in the final analytic sample after prespecified data-quality screening procedures (see Section 2.4).

### 2.2 Procedures

All study materials (participant information sheet, electronic informed consent, and questionnaire) were administered via an online survey platform. The participant information sheet and consent form were displayed on the survey landing page, and only individuals who provided electronic consent were able to proceed to the questionnaire. Survey responses were collected anonymously and stored in a secure database immediately upon submission. Recruitment posters and standardized invitation messages were distributed in January 2023 through student communities and university Facebook groups. Each post included a QR code and survey link. Because recruitment occurred through social media groups, the number of individuals who viewed the invitation could not be determined, which is a recognized limitation of online convenience recruitment. Participants received 150 THB (approximately 5 USD) after verified completion. To maintain anonymity, compensation information was recorded separately from survey responses. Only the research team had access to the password-protected data stored on university servers. The study protocol was conducted in accordance with the Declaration of Helsinki and was approved by the Chiang Mai University Research Ethics Committee (CMUREC No. PSY-2565-0007, date of approval: 18 October 2022).

### 2.3 Measurements

The questionnaire assessed participant demographics, internet use, cyberbullying experiences (cybervictimization and cyberaggression), and self-esteem.

#### 2.3.1 Demographics

Participants reported gender, sexual orientation, age, education level, year of study, faculty, grade point average (GPA), monthly income, and underlying disease status. *Internet use.* Participants reported their average daily time spent using the internet.

#### 2.3.2 Cybervictimization

Cybervictimization was assessed using the Thai version of the Cyberaggression–Perpetration and Victimization Scale (victimization subscale; 12 items; Anuroj and Pitayaratstian, 2019), which was originally developed as the Cyber-Aggression and Cyber-Victimization Scale by Shapka and Maghsoudi (2017). Permission to use the Thai version of the instrument was obtained from Dr. Pityaratstian prior to data collection. The instrument comprises two sections (victimization and perpetration) with 24 items in total. The victimization items cover a range of cyberbullying experiences (e.g., having embarrassing or mean content posted or reposted about oneself, receiving hurtful messages, having an embarrassing photo or video posted, receiving hurtful comments on one’s online photo/video, being purposely excluded online, having personal information posted, having gossip or rumors spread online, receiving hurtful comments about perceived sexual orientation, and receiving unwanted sexual content intended to embarrass). The Thai version was translated and culturally adapted and demonstrated good psychometric properties in the original validation, including strong content validity (item–objective congruence indices 0.80–1.00) and good internal consistency (Cronbach’s α = 0.85 for victimization and 0.856 for perpetration).

In the present study, cybervictimization was operationalized as a behavioral endorsement index to align with the count-based modeling framework. Responses were coded dichotomously (0 = not experienced, 1 = experienced) and summed to represent the number of distinct victimization experiences endorsed. To improve measurement usability in a sparse, zero-heavy distribution, we retained nine victimimization behaviors with sufficient variability for constructing the index, resulting in a final score ranging from 0 to 9, with higher scores indicating endorsement of a greater number of cybervictimization types. Internal consistency for the retained victimization items was Cronbach’s α = 0.75. Item-level endorsement frequencies for the retained items are reported in the Supplementary material.

#### 2.3.3 Cyberaggression (perpetration endorsement count)

Cyberaggression perpetration was assessed using the perpetration item pool from the same instrument (12 items; Anuroj and Pitayaratstian, 2019). Consistent with the outcome definition in the zero-inflated models, perpetration was operationalized as a bounded endorsement-count (behavioral variety) index ranging from 0 to 5. Five perpetration behaviors with sufficient variability were retained and coded dichotomously (0 = no, 1 = yes). Items were summed to yield the cyberaggression endorsement count (AGGRESSO), with higher scores indicating endorsement of a greater number of distinct cyberaggressive behaviors. Internal consistency for the retained items was Cronbach’s α = 0.54. Because this measure is a checklist-style behavioral variety (formative) index of distinct, non-interchangeable behaviors rather than a reflective latent construct, Cronbach’s alpha is of limited interpretability and is reported primarily for transparency. Item-level endorsement frequencies for the retained behaviors are provided in the Supplementary materials to allow readers to evaluate variability and item functioning.

#### 2.3.4 Self-esteem

Self-esteem was assessed using the Thai version of the Rosenberg Self-Esteem Scale. The scale contains *10 items*, each rated from *1 to 4*, yielding a total score range of *10–40*, with higher scores indicating higher self-esteem. The Thai version has demonstrated good reliability (Cronbach’s α = *0.86*; Wongpakaran and Wongpakaran, 2012).

### 2.4 Data screening and preparation

A total of 440 individuals accessed the survey and provided electronic consent. Prespecified data-quality screening procedures were applied to improve data integrity and reproducibility. Exclusion criteria were: (a) unusually short completion time relative to pilot testing (median pilot completion time ≈ 6 min; exclusions < 2 min; *n* = 10), consistent with CHERRIES recommendations for identifying atypically rapid submissions (Eysenbach, 2004); (b) response patterns indicative of straight-lining and/or logical inconsistencies across demographic and behavioral items (*n* = 12); and (c) duplicate submissions identified using submission timestamps, anonymized IP-pattern checks, and compensation records (*n* = 4). No personally identifiable information was retained. Variables were examined for missingness and outliers prior to analysis. For multi-item scales, if at least 80% of items were completed, missing item responses were replaced with the participant’s mean of completed items for that scale, consistent with common psychometric practice. Scale-level missingness was minimal (<1%); therefore, no additional imputation was performed. After screening, the final analytic sample comprised 414 participants.

### 2.5 Statistical analysis

Descriptive statistics were computed to summarize participant characteristics and key study variables. Categorical variables are reported as frequencies and percentages, and continuous variables are reported as medians and interquartile ranges (IQR) due to non-normal distributions. Gender and sexual orientation were included to describe the sample and evaluate generalizability; they were not primary explanatory variables for the study hypotheses.

The primary objective was to examine whether cybervictimization and self-esteem predict cyberaggression endorsement and whether self-esteem moderates the association between cybervictimization and cyberaggression. Variables were inspected for plausible ranges, missingness, and the proportion of zero values. Cybervictimization (VICTIM; 0–9) and self-esteem (ROSENBER) were grand-mean centered prior to computing the interaction term (VICTIM × ROSENBER).

**Figure 1:**
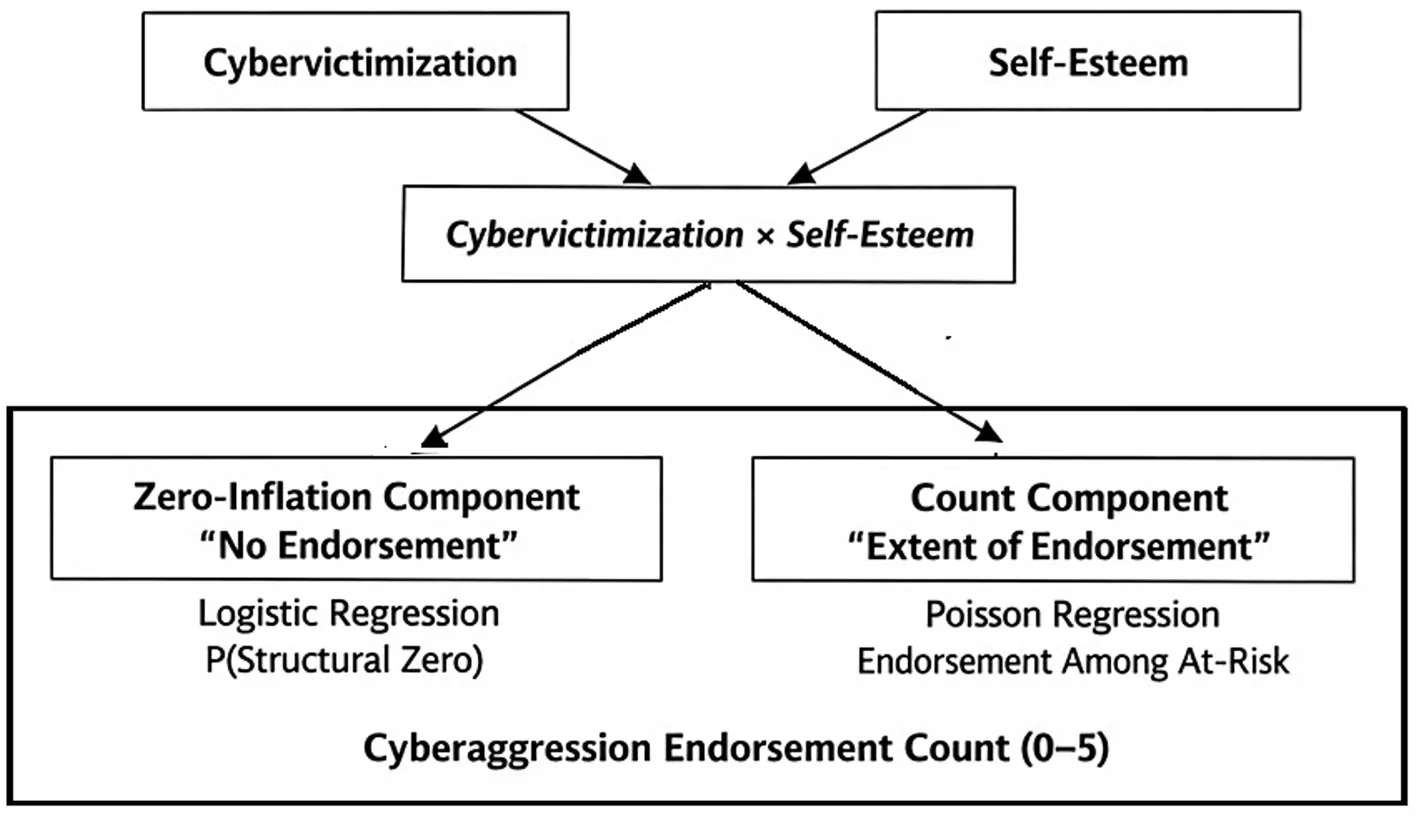
Conceptual framework of cyberaggression endorsement using a zero-inflated Poisson (ZIP) model with moderation by self-esteem. The ZIP model partitions the bounded cyberaggression endorsement count into two simultaneous components: (a) a zero-inflation component, estimated via logistic regression, that models the probability of belonging to a structural-zero/no-endorsement subgroup, and (b) a count component, estimated via Poisson regression, that models the extent of endorsement among individuals at risk. Cybervictimization, self-esteem, and their interaction were specified as predictors in both components to evaluate whether self-esteem moderates associations with (a) the likelihood of any endorsement versus structural non-endorsement, and (b) the degree of endorsement among at-risk individuals.

Cyberaggression (AGGRESSO) was operationalized as a bounded behavioral endorsement count representing the number of distinct cyberaggressive perpetration behaviors endorsed in the past month (range 0–5). Because the outcome exhibited a high proportion of zeros (83.1%) and a positively skewed distribution among non-zero values, we compared Poisson, negative binomial (NB), and zero-inflated Poisson (ZIP) models. Model selection was based on log-likelihood values and information criteria (AIC, BIC, and sample-size adjusted BIC). The primary model was a two-part ZIP model, which estimates (a) a count component representing the expected endorsement count among individuals at risk of endorsing cyberaggressive behaviors and (b) a zero-inflation component representing the probability of belonging to a structural-zero/no-endorsement subgroup (model-implied). The conceptual specification of the ZIP model is shown in Figure 1.

Prior to interpreting model estimates, we evaluated diagnostics relevant to zero-inflated count modeling. AGGRESSO was inspected to confirm a non-negative bounded endorsement count with substantial zero inflation and right-skew. We compared Poisson, NB, and ZIP models using log-likelihood and information criteria (AIC/BIC) and inspected observed versus model-implied frequencies across the 0–5 categories, supporting the ZIP specification. Model convergence and parameter plausibility (e.g., standard errors) were verified. Predictors were grand-mean centered prior to forming the interaction term, and multicollinearity was minimal based on auxiliary-regression VIFs (range = 1.118–1.313).

Cybervictimization, self-esteem, and their interaction were included as predictors in both components of the ZIP model. Effects are reported as unstandardized coefficients with robust standard errors and 95% confidence intervals. Results were interpreted with attention to the direction of the zero-inflation component (i.e., negative coefficients indicate a lower likelihood of belonging to the structural-zero/no-endorsement subgroup, corresponding to a higher likelihood of any endorsement).

#### 2.5.1 Sensitivity analyses

Because AGGRESSO is a bounded endorsement score rather than an unbounded incident count, robustness was evaluated using alternative outcome specifications: (a) a binary logistic regression predicting any cyberaggression endorsement (0 vs. ≥1) and (b) an ordinal logistic regression treating cyberaggression as ordered categories; due to sparse upper categories, values of 3–5 were collapsed into a single category (3+). Consistency of inferences for cybervictimization, self-esteem, and their interaction across the primary ZIP model and the sensitivity models was used to assess robustness.

All analyses were conducted in Mplus version 9.1 using the MLR estimator.

## 3 Results

### 3.1 Descriptive statistics of sample characteristics

A total of 414 university students completed the survey. Participant characteristics are summarized in Table 1. Most participants were female (61.4%) and identified as heterosexual (71.7%). The median age was 20 years (IQR: 19–22), and nearly all participants were enrolled in bachelor’s degree programs (98.3%). Most reported no underlying disease (87.7%), and more than half reported internet use exceeding 6 h per day (54.8%). The median Rosenberg Self-Esteem Scale score was 30 (IQR: 26–33). With respect to cyberbullying-related experiences, 78.5% reported ever experiencing cyberbullying, 33.8% reported victimization experiences, and 16.9% reported perpetration experiences. Cyberaggression endorsement was highly zero-inflated (83.1% zeros; range 0–5), motivating evaluation of zero-inflated count models.

**Table 1:** Characteristics of the participants (*N* = 414).

Demographic (n (%) or Median [IQR])	All (*N* = 414)
Gender	
Male	145 (35.0%)
Female	254 (61.4%)
Other	15 (3.6%)
Sexual orientation	
Heterosexual	297 (71.7%)
LGBTQIA+	117 (28.3%)
Age	20 [19–22]
Education	
Bachelor’s	407 (98.3%)
Postgraduate	7 (1.7%)
Year	
1st	107 (25.8%)
2nd	116 (28.0%)
3rd	52 (12.6%)
4th	47 (11.4%)
5th	26 (6.3%)
6th	59 (14.3%)
Postgraduate	7 (1.7%)
Faculty	
Health sciences	240 (58.0%)
Natural and technological sciences	27 (6.5%)
Humanities and social sciences	147 (35.5%)
GPA	
<2.50	28 (6.8%)
2.50–2.99	93 (22.7%)
3.00–3.49	144 (35.1%)
3.50–4.00	145 (35.4%)
Income/month (USD)	
0–150	88 (21.3%)
151–300	202 (48.8%)
301–500	90 (21.7%)
>500	34 (8.2%)
Underlying disease	
No	363 (87.7%)
Yes	51 (12.3%)
Internet usage	
<5 h./d	54 (13.0%)
5–6 h./d	133 (32.1%)
>6 h./d	227 (54.8%)
Cyberbullying experience	
Never	89 (21.5%)
Ever	325 (78.5%)
Rosenberg self-esteem scale	30 [26–33]
Cyberbullying victimization experience	
Never	274 (66.2%)
Ever	140 (33.8%)
Any cyberaggression endorsement (AGGRESSO ≥ 1)	
Never	344 (83.1%)
Ever	70 (16.9%)

Additional participant characteristics are presented in Table 1.

Supplementary Table S1 presents Pearson correlations among the study variables. Cybervictimization was positively associated with cyberaggression endorsement (*r* = 0.33, *p* < 0.01). Self-esteem (Rosenberg total) was negatively associated with cybervictimization (*r* = −0.23, *p* < 0.01) and with cyberaggression endorsement (*r* = −0.14, *p* < 0.01). Internet use was negatively associated with age (*r* = −0.21, *p* < 0.01). Other bivariate associations were small. These bivariate patterns are consistent with the hypothesized links among victimization, self-esteem, and cyberaggression, although multivariable ZIP models were used for primary inference.

### 3.2 Model comparison

Model fit comparisons are presented in Table 2. Relative fit improved from the standard Poisson model to the negative binomial (NB) model and was best for the zero-inflated Poisson (ZIP) model. The ZIP model had the highest loglikelihood (H0 = −234.072) and the lowest AIC (484.143), BIC (516.350), and sample-size adjusted BIC (490.965), consistent with substantial excess zeros in the cyberaggression endorsement count.

**Table 2:** Model comparison.

Model	Free parameters	Loglikelihood (H0)	AIC	BIC	Sample-size adjusted BIC
Poisson	4	−266.916	541.832	557.935	545.242
Negative binomial (NB)	5	−244.357	498.714	518.843	502.977
Zero-inflated Poisson (ZIP)	8	−234.072	484.143	516.350	490.965

Lower AIC/BIC/aBIC indicates better relative fit. Across candidate count models, fit improved from Poisson to NB and was best for the ZIP model (lowest AIC/BIC/aBIC), consistent with substantial excess zeros in the cyberaggression endorsement score.

### 3.3 Zero-inflated Poisson (zip) model

The ZIP model estimates two components: (a) a count component, representing the expected cyberaggression endorsement count among individuals at risk of endorsing cyberaggressive behaviors, and (b) a zero-inflation component, representing the probability of belonging to a structural-zero/no-endorsement subgroup. Full model estimates are provided in Table 3.

**Table 3:** Regression results: zero-inflated Poisson (ZIP) model predicting cyberaggression endorsement count (0–5).

Component	Predictor	Estimate (b)	SE	Est./SE	*p*-value	Effect size
Count component (extent among at-risk)	Cybervictimization (VICTIM)	0.048	0.055	0.867	0.386	IRR = 1.049
	Self-esteem (ROSENBER)	−0.078	0.028	−2.812	0.005	IRR = 0.925
	Interaction (VICTIM × ROSENBER)	0.015	0.011	1.342	0.180	IRR = 1.015
Zero-inflation component (structural zero / no endorsement)	Cybervictimization (VICTIM)	−1.406	0.729	−1.928	0.054	OR = 0.245
	Self-esteem (ROSENBER)	−0.057	0.058	−0.995	0.320	OR = 0.945
	Interaction (VICTIM × ROSENBER)	−0.106	0.054	−1.958	0.050	OR = 0.899
Intercepts	Zero-inflation intercept (AGGRESSO#1)	0.591	0.537	1.101	0.271	OR = 1.806
	Count intercept (AGGRESSO)	−0.232	0.215	−1.079	0.281	IRR = 0.793

Cyberaggression (AGGRESSO) was modeled as a bounded endorsement count with a count component (extent of endorsed cyberaggressive behaviors among at-risk individuals) and a zero-inflation component (probability of belonging to a structural-zero/no-endorsement group). Predictors (cybervictimization, self-esteem, and their interaction) were grand-mean centered prior to forming the interaction term. Unstandardized coefficients are reported with robust standard errors (MLR). Negative coefficients in the zero-inflation component indicate a lower likelihood of belonging to the structural-zero group (i.e., higher likelihood of any endorsement). For the count component, effect sizes are incidence rate ratios (IRR = exp[b]). For the zero-inflation component, effect sizes are odds ratios (OR = exp[b]) for membership in the structural-zero/no-endorsement subgroup; thus, OR < 1 indicates a lower likelihood of structural zero (i.e., a higher likelihood of any endorsement).

#### 3.3.1 Count component (extent of endorsement among at-risk individuals)

In the count component, Self-esteem was significantly negatively associated with endorsement extent (*b* = −0.078, *p* = 0.005; IRR = 0.925). This indicates that, among at-risk individuals, each one-unit increase in self-esteem was associated with an estimated 7.5% lower expected cyberaggression endorsement count. Cybervictimization was not associated with the extent of endorsement (*b* = 0.048, *p* = 0.386; IRR = 1.049). Among those at risk, a one-unit increase in victimization corresponded to an estimated 4.9% higher expected endorsement count, but this effect was not statistically reliable. The victimization × self-esteem interaction was not significant for endorsement extent (*b* = 0.015, *p* = 0.180; IRR = 1.015), suggesting no clear evidence that self-esteem moderates victimization effects on how many behaviors are endorsed once individuals are already in the at-risk group.

#### 3.3.2 Zero-inflation component (structural zero/no endorsement)

**Figure 2:**
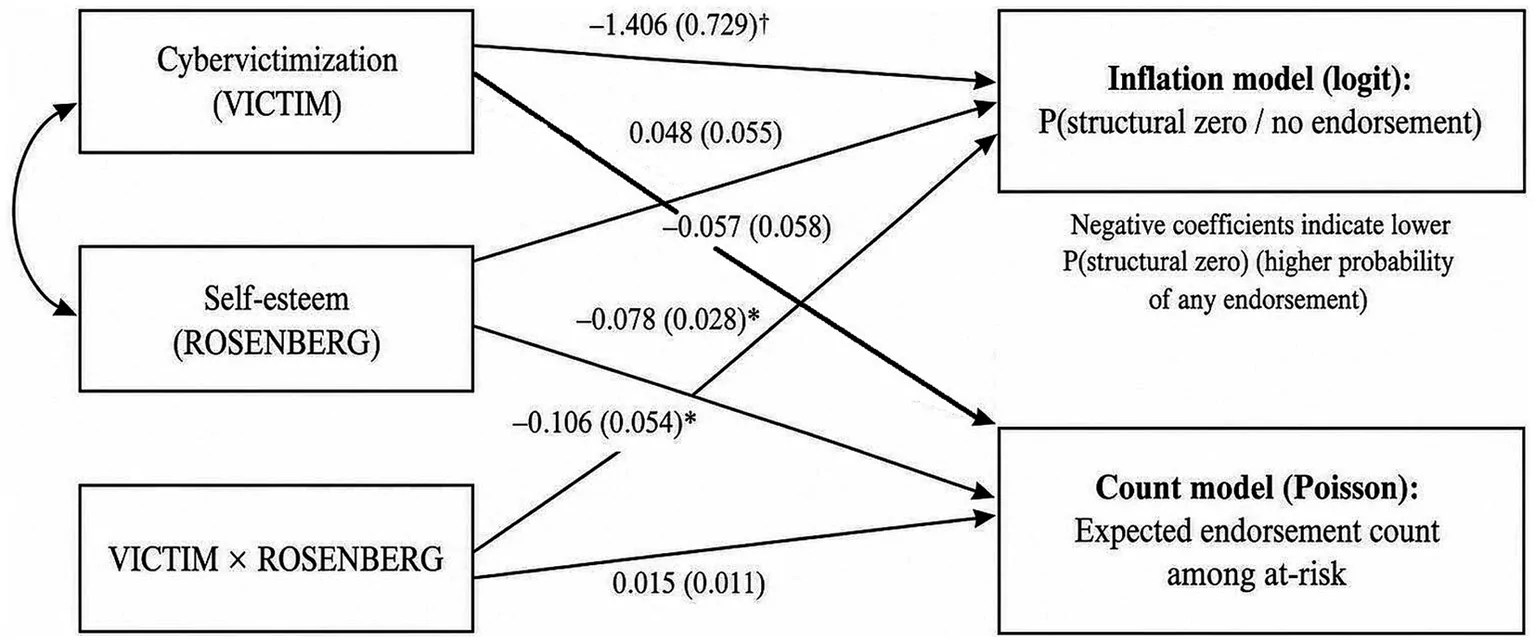
Path diagram of the zero-inflated Poisson (ZIP) regression model predicting cyberaggression endorsement (0–5). Unstandardized coefficients are shown with robust standard errors in parentheses (MLR). The model includes a zero-inflation component estimating the probability of belonging to the structural-zero/no-endorsement subgroup and a count component estimating endorsement extent among at-risk individuals. Cybervictimization, self-esteem, and their interaction were entered as predictors in both components. Negative coefficients in the zero-inflation component indicate a lower likelihood of belonging to the structural-zero subgroup (i.e., a higher likelihood of any endorsement).

Cybervictimization showed a marginal association with moving away from structural non-endorsement (*b* = −1.406, *p* = 0.054; OR = 0.245). This implies that higher victimization was associated with substantially lower odds of being a structural non-endorser, i.e., a higher likelihood of any cyberaggression endorsement, though this effect narrowly missed conventional significance. Self-esteem was not significantly related to structural zero status (*b* = −0.057, *p* = 0.320; OR = 0.945). The victimization × self-esteem interaction was statistically significant (*b* = −0.106, *p* = 0.050; OR = 0.899) (see Figure 2). This indicates that self-esteem moderated the victimization–involvement association: as self-esteem increases, the association between victimization and *reduced* likelihood of structural non-endorsement becomes stronger—i.e., the victimization-related likelihood of any cyberaggression endorsement increases more steeply at higher self-esteem levels.

**Figure 3:**
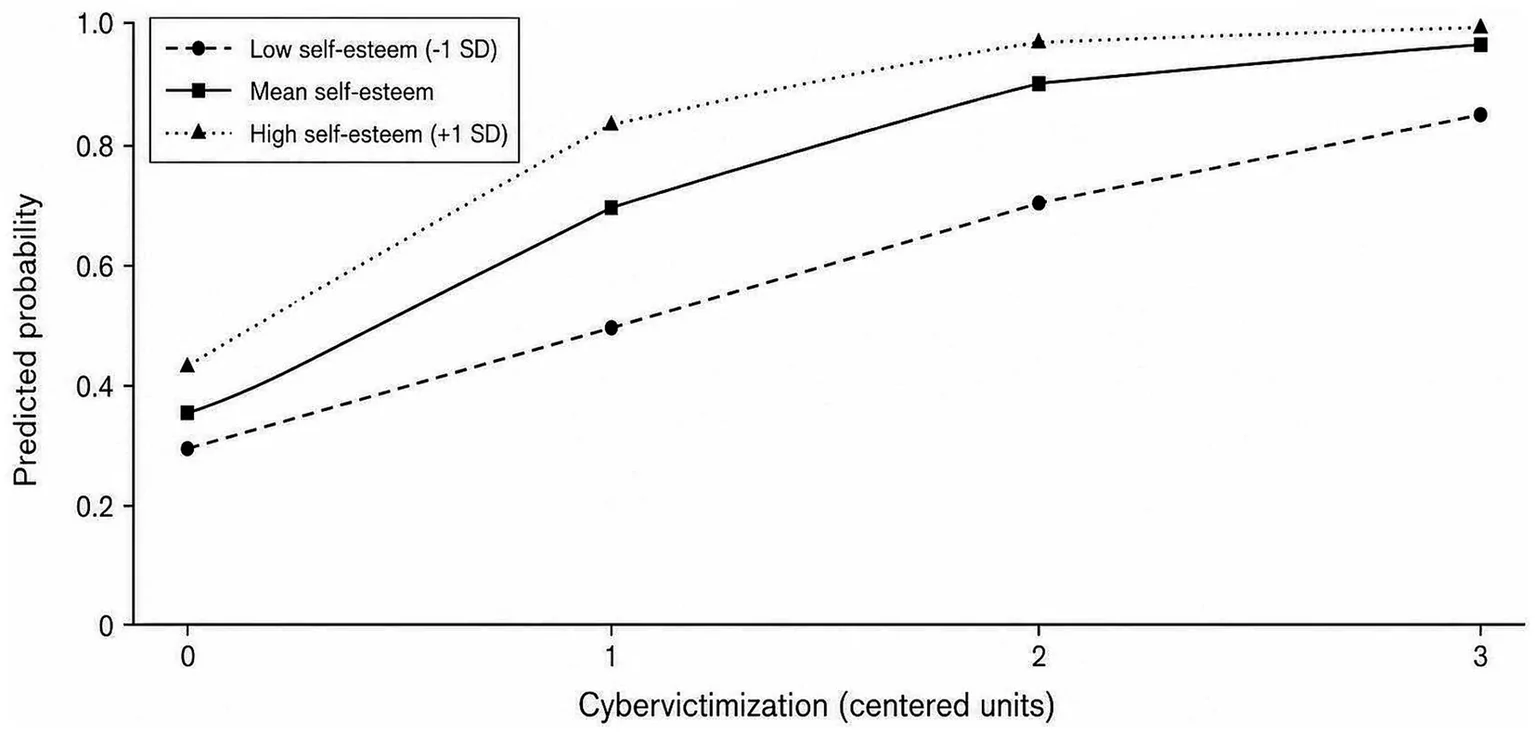
Predicted probability of any cyberaggression endorsement (AGGRESSO ≥ 1). Figure presents model-implied predicted probabilities of any cyberaggression endorsement (AGGRESSO ≥ 1) derived from the ZIP inflation component across levels of cybervictimization at low (−1 SD), mean, and high (+1 SD) self-esteem. Predicted probabilities increased with cybervictimization at all self-esteem levels, indicating that greater victimization was associated with a higher likelihood of any endorsement. The lines were clearly separated, such that predicted probabilities were consistently higher at higher self-esteem, consistent with moderation of the involvement (any vs. none) process by self-esteem (i.e., a victimization × self-esteem interaction in the inflation component).

In the primary ZIP model, self-esteem was significantly associated with lower cyberaggression endorsement extent in the count component (*b* = −0.078, *p* = 0.005), supporting H2. In the zero-inflation component, the cybervictimization × self-esteem interaction was at the conventional α = 0.05 threshold (*b* = −0.106, *p* = 0.050), suggesting moderation of the involvement (any-endorsement vs. structural non-endorsement) process; the main effect of cybervictimization was marginal (*p* = 0.054). Importantly, this moderation effect remained statistically significant in the covariate-adjusted sensitivity model (Supplementary Table S2; *p* = 0.006). A simple-slopes probe of the inflation component (Table 4) indicated that cybervictimization significantly decreased the odds of structural non-endorsement at low, mean, and high self-esteem, consistent with the predicted-probability patterns in Figure 3.

**Table 4:** Simple slopes of cybervictimization predicting structural-zero membership (ZIP inflation component) at low, mean, and high self-esteem.

Self-esteem level	Simple slope *b* (SE)	*z*	*p*	OR = exp.(b)	95% CI for OR*
Low (−1 SD)	−1.049 (0.348)	−3.010	0.003	0.350	[0.177, 0.693]
Mean (0)	−1.661 (0.523)	−3.174	0.002	0.190	[0.068, 0.529]
High (+1 SD)	−2.274 (0.725)	−3.139	0.002	0.103	[0.025, 0.426]

Slopes are from the inflation (structural-zero) logit. ORs refer to the odds of being a structural zero (no endorsement); thus OR < 1 indicates lower odds of structural non-endorsement (higher probability of any endorsement). *95% CI for OR computed as exp (b ± 1.96 × SE), i.e., by exponentiating the 95% CI of the log-odds slope.

Covariate-adjusted sensitivity analysis. To evaluate robustness to established correlates, we re-estimated the ZIP model including age, gender (male = 1, female = 0), and internet-use level as covariates in both the count and inflation components. The substantive pattern of findings was unchanged. In the count component, higher self-esteem remained associated with a lower extent of cyberaggression endorsement among at-risk individuals (*b* = −0.083, *p* = 0.009), whereas cybervictimization and the cybervictimization × self-esteem interaction was not statistically significant. In the inflation component, cybervictimization was associated with lower odds of structural non-endorsement (*b* = −1.661, *p* = 0.002), and the cybervictimization × self-esteem interaction remained statistically significant (*b* = −0.122, *p* = 0.006), supporting the moderation interpretation shown in Figure 3. Among covariates, age was positively associated with structural non-endorsement (*b* = 1.012, *p* = 0.022). Full covariate-adjusted estimates are reported in Supplementary Table S2.

### 3.4 Sensitivity analyses

Sensitivity analyses supported the robustness of the primary findings under alternative outcome specifications. In a binary logistic regression predicting any cyberaggression endorsement (0 vs. ≥ 1), cybervictimization was associated with higher odds of endorsement (*b* = 0.626, SE = 0.101, *p* < 0.001; OR = 1.871, 95% CI [1.535, 2.280]), whereas self-esteem was not significant (*b* = −0.030, SE = 0.030, *p* = 0.327). The cybervictimization × self-esteem interaction remained significant (*b* = 0.052, SE = 0.016, *p* = 0.001; OR = 1.053, 95% CI [1.021, 1.087]). Similarly, an ordinal logistic regression treating cyberaggression as ordered categories (0, 1, 2, 3+) showed that cybervictimization remained significant (*b* = 0.544, SE = 0.103, *p* < 0.001; OR = 1.722, 95% CI [1.408, 2.107]), self-esteem remained non-significant (*b* = −0.036, SE = 0.028, *p* = 0.198), and the interaction remained significant (*b* = 0.035, SE = 0.016, *p* = 0.027; OR = 1.036, 95% CI [1.004, 1.069]). Overall, these models were consistent with the interpretation that cybervictimization (and its moderation by self-esteem) is most evident for cyberaggression involvement (any endorsement), whereas self-esteem shows its clearest association with endorsement extent in the primary ZIP count component. These sensitivity models mirror the two-part ZIP pattern: victimization and the interaction primarily differentiate “any vs. none,” while self-esteem is more strongly related to the frequency/extent among those with any endorsement.

## 4 Discussion

Against the backdrop of increasing concern about cybervictimization and cyberaggression among university students—and limited evidence from the Thai context—the present study examined whether cybervictimization was associated with cyberaggression endorsement and whether self-esteem moderated this association using a two-part zero-inflated Poisson (ZIP) framework. Overall, the findings suggest that correlates of cyberaggression involvement (i.e., movement away from structural non-endorsement) may differ from correlates of the extent of endorsement among individuals who are already at risk of endorsing cyberaggressive behaviors. Sensitivity analyses using binary and ordinal specifications further supported the robustness of the victimization association and the victimization × self-esteem interaction for the involvement aspect of cyberaggression.

### 4.1 Cybervictimization and cyberaggression endorsement (H1)

H1 proposed that cybervictimization would be positively associated with cyberaggression. Results provided partial support. In the ZIP model, cybervictimization was not significantly associated with the count component (extent of endorsement among at-risk individuals), but its association with the zero-inflation component was marginal (*p* = 0.054), and the sensitivity analyses indicated a clear positive association between cybervictimization and the likelihood of any endorsement. This pattern is broadly consistent with prior work documenting positive links between cybervictimization and cyberaggression and with longitudinal evidence suggesting that victimization can precede later aggression rather than merely co-occur with it (Camacho et al., 2021; Wright and Li, 2013; Zhang et al., 2025). At the same time, the two-part results qualify the victimization–aggression link by suggesting that cybervictimization may be more strongly related to whether individuals become involved in cyberaggression (any endorsement) than to how many cyberaggressive behaviors they endorse once involved.

A plausible interpretation is that victimization experiences may increase the probability of crossing an “involvement threshold” (e.g., by increasing anger, stress, or perceived justification for retaliation), whereas the extent of subsequent endorsement may depend more on proximal self-regulatory processes and situational opportunities (Ak et al., 2015; Camacho et al., 2021; Luo et al., 2023; Quintana-Orts et al., 2020). This distinction is important because it implies that prevention may need to focus not only on reducing repeated perpetration but also on preventing escalation from victimization to initial involvement.

### 4.2 Self-esteem as a moderator (H2a/H2b)

The study further examined whether self-esteem moderated the association between cybervictimization and cyberaggression. Moderation was supported primarily for the involvement-related process. In the ZIP model, the cybervictimization × self-esteem interaction was statistically significant in the zero-inflation component (*p* = 0.050), whereas the interaction was not significant in the count component. The binary and ordinal sensitivity models showed the same general pattern, with significant interactions predicting any endorsement and ordered endorsement categories. Overall, these results indicate that self-esteem conditioned the association between cybervictimization and movement away from structural non-endorsement, but not the extent of endorsement among those already at risk.

Substantively, the interaction suggests that the victimization–involvement association differs by self-esteem level. One way to interpret this pattern is that cybervictimization may carry self-relevant meaning (e.g., rejection, humiliation, or diminished social worth) and that self-esteem influences how strongly such experiences translate into behavioral responding. Self-esteem is embedded in interpersonal functioning and sensitivity to social evaluation (Harris and Orth, 2020; Orth et al., 2018), and is closely related to shame and broader psychological well-being (Budiarto and Helmi, 2021; Muris and Otgaar, 2023). Cybervictimization can convey negative signals about social value (Kim and Lee, 2020). From this perspective, moderation may emerge more clearly at the “gatekeeping” stage (any endorsement vs. none) because initial involvement may be more sensitive to how victimization is appraised and internalized.

At the same time, self-esteem showed a clear direct association with the count component: higher self-esteem predicted a lower extent of cyberaggression endorsement among at-risk individuals. This finding suggests that self-esteem may function as a protective resource once individuals are in the risk pool for endorsement, potentially by supporting more adaptive coping or reducing escalation after initial involvement. Importantly, the present results do not imply that a structural-zero subgroup represents “always non-aggressors” in an absolute sense. Rather, the structural-zero/no-endorsement component is a model-based distinction that reflects heterogeneous reasons for zero endorsement (e.g., low propensity/opportunity, strong norms against aggression, or underreporting). Therefore, interpretation of this component should remain cautious and probabilistic.

The form of the interaction suggests that cybervictimization was more strongly related to *any* cyberaggression endorsement at higher levels of self-esteem. One possible interpretation is that, for some students, higher self-esteem may be associated with a greater tendency to respond to provocation in a self-protective or retaliatory manner (e.g., defending one’s social standing, “pushing back,” or restoring perceived respect following public online humiliation). In this view, victimization may function as an ego-relevant or status-relevant threat, and students with higher self-esteem may be more likely to engage in at least one cyberaggressive response when they feel wronged. Importantly, this interpretation is speculative and should be evaluated in future research using longitudinal designs and mediators such as anger, revenge motivation, perceived provocation, and normative beliefs about retaliation. Alternative explanations are also plausible (e.g., differences in opportunity, peer context, social dominance orientation, or reporting tendencies), and therefore the present findings should be interpreted as conditional associations rather than evidence of a protective mechanism operating through aggression. At the same time, higher self-esteem was associated with *lower extent* of cyberaggression endorsement among at-risk individuals in the ZIP count component, suggesting that even if higher self-esteem is linked to crossing the involvement threshold under provocation, it may still function as a resource that reduces escalation or repetition once involved.

### 4.3 Integrating mechanisms and theory

This component-specific pattern can be interpreted through Sociometer Theory, which conceptualizes self-esteem as an internal gauge of perceived relational value and social inclusion. This interpretation is also consistent with evidence that shame is associated with lower self-esteem (Budiarto and Helmi, 2021), although shame was not measured in the present study and should be examined directly in future research. Cybervictimization may signal rejection or devaluation, and such cues may be especially consequential for behavior at the involvement “gatekeeping” stage (whether one engages at all). In addition, threatened egotism perspectives suggest that ego-relevant provocation can prompt retaliatory or defensive responses under some conditions; thus, the victimization × self-esteem interaction in the involvement process may reflect differences in how victimization is appraised as a self-relevant threat (Leary, 2005). By contrast, the extent of endorsement among already at-risk individuals may depend more on downstream processes such as emotion regulation, rumination, and opportunity structures, which may explain why moderation did not emerge in the count component.

Prior research indicates that the pathway from cybervictimization to cyberaggression is unlikely to be purely direct and may involve anger, stress, rumination, and revenge-related motives (Ak et al., 2015; Camacho et al., 2021; Luo et al., 2023; Quintana-Orts et al., 2020). The present component-specific pattern is compatible with the idea that victimization may increase the likelihood of involvement through heightened negative affect and threat appraisals, whereas endorsement extent may be shaped by downstream regulatory processes that govern persistence and escalation (e.g., rumination and maladaptive coping). In addition, review evidence suggests that cybervictimization is associated with broader self-related constructs beyond self-esteem, including self-efficacy and self-concept (Anichitoae et al., 2025). This broader self-evaluative profile may be particularly relevant for understanding why self-esteem moderated involvement but not extent in the ZIP model.

### 4.4 Theoretical and methodological implications

The findings underscore that cyberaggression endorsement should not be treated as a unitary outcome. Many studies model cyberaggression as a single process (often dichotomous involvement), which can obscure potentially different predictors of (a) involvement and (b) endorsement extent among those involved. By applying a two-part zero-inflated framework to a bounded, zero-heavy endorsement count, the present study demonstrates that cybervictimization and self-esteem may operate differently across these components. Thus, the two-part model was not only a statistical accommodation to the distributional properties of the outcome, but also a theoretically informative framework that distinguishes predictors of initial involvement from predictors of escalation/extent among at-risk individuals.

The findings also refine how self-esteem is understood in cyberbullying literature. Rather than functioning as a uniform “buffer” across all aspects of cyberaggression, self-esteem showed a differentiated pattern: a direct negative association with endorsement extent among at-risk individuals, alongside a conditional role (via interaction with victimization) for the involvement-related process. This distinction may help reconcile mixed prior findings by suggesting that self-esteem’s relevance depends on the aspect of cyberaggression being modeled.

### 4.5 Practical implications

The results have implications for prevention and support in university settings. First, interventions may benefit from targeting not only students who repeatedly endorse cyberaggressive behaviors but also students who experience cybervictimization and may be vulnerable to becoming involved in cyberaggression. If victimization is more strongly associated with involvement than with endorsement extent, timely support following victimization—such as accessible reporting channels, peer support, and skills for adaptive coping—may help prevent escalation into perpetration.

Second, self-esteem may represent a useful intervention target, but it should not be viewed in isolation. Reviews suggest that self-esteem can be improved through psychological interventions, particularly cognitive-behavioral approaches, although typical effects are small to moderate (Kolubinski et al., 2018). Given evidence implicating anger, rumination, and revenge motivations in victimization-to-aggression pathways (Ak et al., 2015; Camacho et al., 2021; Luo et al., 2023; Quintana-Orts et al., 2020), skills-based components that strengthen emotion regulation and reduce maladaptive coping may be especially relevant in campus programming. Mindfulness-based approaches may also be considered as an adjunct, given evidence linking such approaches to reductions in self-critical thinking and improvements in self-esteem (Randal et al., 2015), although these mechanisms were not tested directly in the present study.

In university settings, these findings support multi-component approaches that combine (a) clear reporting pathways and rapid support following victimization, (b) prevention content addressing online norms and bystander responding (e.g., digital citizenship and peer-led campaigns), and (c) counseling or skills-based programs targeting coping with provocation, emotion regulation, and rumination. Such components may be particularly relevant if victimization is most strongly associated with the transition from non-endorsement to initial involvement, while escalation among involved students is more closely tied to self-regulatory and situational factors.

### 4.6 Limitations and future direction

Several limitations should be acknowledged. First, the cross-sectional design precludes causal inference and does not establish temporal ordering among cybervictimization, self-esteem, and cyberaggression. Although the observed associations are consistent with the possibility that self-esteem conditions responses to cybervictimization, the reverse direction is also plausible (e.g., repeated harmful online experiences may erode self-esteem over time), and bidirectional processes cannot be ruled out. Future studies should use longitudinal and/or intensive repeated-measures designs (e.g., multiple waves across a semester or ecological momentary assessment) to test temporal precedence, reciprocal pathways, and lagged moderation effects. Second, all constructs were assessed via self-report, which may introduce shared method variance and response bias. In particular, cyberaggression may be underreported due to social desirability concerns, and such underreporting could contribute to excess zeros. Relatedly, the structural-zero/no-endorsement component of the ZIP model is a model-based distinction rather than a directly observed subgroup; therefore, it should not be interpreted as indicating “always non-aggressors,” and alternative explanations for zero endorsement (e.g., low opportunity/exposure, strong anti-aggression norms, or reluctance to disclose) remain possible. Future research should incorporate multi-method assessment, such as peer reports, behavioral indicators (where ethically feasible), indirect questioning techniques, or validity checks for underreporting, and should examine whether the zero-inflation component aligns with measured opportunity/norms or disclosure reluctance. Third, participants were recruited via online convenience sampling from a single university (Chiang Mai University), which was a feasibility- and anonymity-oriented design choice for sensitive self-report outcomes. However, this approach may introduce selection bias (e.g., overrepresentation of students who are more active in online groups) and limits external validity, particularly given that the sample was predominantly female. Future studies should replicate these findings using larger, multi-university samples across regions of Thailand and, where feasible, probability-based or stratified recruitment approaches, and should test whether key parameters differ across demographic groups (e.g., gender and sexual orientation) using adequately powered subgroup or moderation analyses. Fourth, cyberaggression was operationalized as a bounded behavioral endorsement count (0–5) with a high proportion of zeros. While the ZIP approach was appropriate for these distributional features and was supported by model comparison and sensitivity analyses, the limited range may reduce precision for distinguishing low-frequency involvement from non-involvement. Future research could use more fine-grained measurement (e.g., expanded item coverage, frequency response options, event-based designs, or repeated assessments) to better separate true non-involvement from low-rate involvement and to evaluate whether the same component-specific pattern holds with richer measures. Fifth, the interpretation draws on mechanisms such as shame/self-critical processing, anger, rumination, stress, and coping, but these processes were not measured directly. Future studies should include measured mediators and moderators (e.g., anger/rumination scales, coping style, perceived norms, emotion regulation, and revenge motivation) to test the proposed mechanisms and to clarify why self-esteem appears more relevant to the involvement (zero-inflation) process than to endorsement extent among at-risk individuals.

## 5 Conclusion

Cybervictimization and cyberaggression are growing concerns among university students, yet evidence from Thailand remains limited. Using a two-part zero-inflated Poisson (ZIP) approach to model a bounded, zero-heavy cyberaggression endorsement count, the present study suggests that cybervictimization is more closely related to cyberaggression involvement (movement away from structural non-endorsement) than to the extent of endorsement among individuals already at risk. Self-esteem was directly associated with lower endorsement extent in the count component, while its moderating role was most evident for the involvement-related (zero-inflation) process in interaction with cybervictimization. Overall, these findings highlight the value of distinguishing involvement from endorsement extent when studying cyberaggression and suggest that university prevention efforts may benefit from timely support following victimization alongside interventions that strengthen self-regulation and adaptive coping.

## Data Availability

The raw data supporting the conclusions of this article will be made available by the authors, without undue reservation.
