# Supplementary material for: Cybervictimization, self-esteem, and cyberaggression endorsement in Thai university students: evidence from a two-part zero-inflated model
Source: Front Psychol. 2026 Sep 11;17:1863842. doi: 10.3389/fpsyg.2026.1863842 (PMC13612487; doi:10.3389/fpsyg.2026.1863842)
Supplement: Supplementary file 1 [file Data_Sheet_1.PDF]

Supplementary File

CB_01_v

		Frequency	Percent	Valid Percent	Cumulative Percent
Valid	0	386	75.1	93.2	93.2
	1	28	5.4	6.8	100.0
	Total	414	80.5	100.0	

CB_01_a

		Frequency	Percent	Valid Percent	Cumulative Percent
Valid	0	412	80.2	99.5	99.5
	1	2	.4	.5	100.0
	Total	414	80.5	100.0	

CB_02_v

		Frequency	Percent	Valid Percent	Cumulative Percent
Valid	0	339	66.0	81.9	81.9
	1	75	14.6	18.1	100.0
	Total	414	80.5	100.0	

CB_02_a

		Frequency	Percent	Valid Percent	Cumulative Percent
Valid	0	386	75.1	93.2	93.2
	1	28	5.4	6.8	100.0
	Total	414	80.5	100.0	

CB_03_v

		Frequency	Percent	Valid Percent	Cumulative Percent
Valid	0	381	74.1	92.0	92.0
	1	33	6.4	8.0	100.0
	Total	414	80.5	100.0	

CB_03_a

		Frequency	Percent	Valid Percent	Cumulative Percent
Valid	0	404	78.6	97.6	97.6
	1	10	1.9	2.4	100.0
	Total	414	80.5	100.0	

CB_04_v

		Frequency	Percent	Valid Percent	Cumulative Percent
Valid	0	379	73.7	91.5	91.5
	1	35	6.8	8.5	100.0
	Total	414	80.5	100.0	

CB_04_a

		Frequency	Percent	Valid Percent	Cumulative Percent
Valid	0	398	77.4	96.1	96.1
	1	16	3.1	3.9	100.0
	Total	414	80.5	100.0	

CB_05_v

		Frequency	Percent	Valid Percent	Cumulative Percent
Valid	0	394	76.7	95.2	95.2
	1	20	3.9	4.8	100.0
	Total	414	80.5	100.0	
Missing	System	100	19.5		
Total		514	100.0		

CB_05_a

		Frequency	Percent	Valid Percent	Cumulative Percent
Valid	0	409	79.6	98.8	98.8
	1	5	1.0	1.2	100.0
	Total	414	80.5	100.0	

CB_06_v

		Frequency	Percent	Valid Percent	Cumulative Percent
Valid	0	379	73.7	91.5	91.5
	1	35	6.8	8.5	100.0
	Total	414	80.5	100.0	

CB_06_a

		Frequency	Percent	Valid Percent	Cumulative Percent
Valid	0	413	80.4	99.8	99.8
	1	1	.2	.2	100.0
	Total	414	80.5	100.0	

CB_07_v

		Frequency	Percent	Valid Percent	Cumulative Percent
Valid	0	395	76.8	95.4	95.4
	1	19	3.7	4.6	100.0
	Total	414	80.5	100.0	

CB_07_a

		Frequency	Percent	Valid Percent	Cumulative Percent
Valid	0	412	80.2	99.5	99.5
	1	2	.4	.5	100.0
	Total	414	80.5	100.0	

CB_08_v

		Frequency	Percent	Valid Percent	Cumulative Percent
Valid	0	406	79.0	98.1	98.1
	1	8	1.6	1.9	100.0
	Total	414	80.5	100.0	

CB_08_a

		Frequency	Percent	Valid Percent	Cumulative Percent
Valid	0	410	79.8	99.0	99.0
	1	4	.8	1.0	100.0
	Total	414	80.5	100.0	

CB_09_v

		Frequency	Percent	Valid Percent	Cumulative Percent
Valid	0	381	74.1	92.0	92.0
	1	33	6.4	8.0	100.0
	Total	414	80.5	100.0	

CB_09_a

		Frequency	Percent	Valid Percent	Cumulative Percent
Valid	0	409	79.6	98.8	98.8
	1	5	1.0	1.2	100.0
	Total	414	80.5	100.0	

CB_10_v

		Frequency	Percent	Valid Percent	Cumulative Percent
Valid	0	410	79.8	99.0	99.0
	1	4	.8	1.0	100.0
	Total	414	80.5	100.0	

CB_10_a

		Frequency	Percent	Valid Percent	Cumulative Percent
Valid	0	410	79.8	99.0	99.0
	1	4	.8	1.0	100.0
	Total	414	80.5	100.0	

CB_11_v

		Frequency	Percent	Valid Percent	Cumulative Percent

Valid	0	398	77.4	96.1	96.1
	1	16	3.1	3.9	100.0
	Total	414	80.5	100.0	

CB_11_a

		Frequency	Percent	Valid Percent	Cumulative Percent
Valid	0	400	77.8	96.6	96.6
	1	14	2.7	3.4	100.0
	Total	414	80.5	100.0	

CB_12_v

		Frequency	Percent	Valid Percent	Cumulative Percent
Valid	0	383	74.5	92.5	92.5
	1	31	6.0	7.5	100.0
	Total	414	80.5	100.0	

CB_12_a

		Frequency	Percent	Valid Percent	Cumulative Percent
Valid	0	391	76.1	94.4	94.4
	1	23	4.5	5.6	100.0
	Total	414	80.5	100.0	
