# Supplementary material for: Cybervictimization, self-esteem, and cyberaggression endorsement in Thai university students: evidence from a two-part zero-inflated model
Source: Front Psychol. 2026 Sep 11;17:1863842. doi: 10.3389/fpsyg.2026.1863842 (PMC13612487; doi:10.3389/fpsyg.2026.1863842)
Supplement: Supplementary file 2 [file Table_1.docx]

| **Table S1. CB Variables Frequency Distribution (v = Victim, a = Abuser)** | | | | | | |
| --- | --- | --- | --- | --- | --- | --- |
| **Variable** | **Role** | **Value** | **Frequency** | **Percent** | **Valid Percent** | **Cumulative Percent** |
| **CB_01_v** | Victim | 0 | 386 | 75.1 | 93.2 | 93.2 |
|  |  | 1 | 28 | 5.4 | 6.8 | 100 |
| **CB_01_a** | Abuser | 0 | 412 | 80.2 | 99.5 | 99.5 |
|  |  | 1 | 2 | 0.4 | 0.5 | 100 |
| **CB_02_v** | Victim | 0 | 339 | 66 | 81.9 | 81.9 |
|  |  | 1 | 75 | 14.6 | 18.1 | 100 |
| **CB_02_a** | Abuser | 0 | 386 | 75.1 | 93.2 | 93.2 |
|  |  | 1 | 28 | 5.4 | 6.8 | 100 |
| **CB_03_v** | Victim | 0 | 381 | 74.1 | 92 | 92 |
|  |  | 1 | 33 | 6.4 | 8 | 100 |
| **CB_03_a** | Abuser | 0 | 404 | 78.6 | 97.6 | 97.6 |
|  |  | 1 | 10 | 1.9 | 2.4 | 100 |
| **CB_04_v** | Victim | 0 | 379 | 73.7 | 91.5 | 91.5 |
|  |  | 1 | 35 | 6.8 | 8.5 | 100 |
| **CB_04_a** | Abuser | 0 | 398 | 77.4 | 96.1 | 96.1 |
|  |  | 1 | 16 | 3.1 | 3.9 | 100 |
| **CB_05_v** | Victim | 0 | 394 | 76.7 | 95.2 | 95.2 |
|  |  | 1 | 20 | 3.9 | 4.8 | 100 |
| **CB_05_a** | Abuser | 0 | 409 | 79.6 | 98.8 | 98.8 |
|  |  | 1 | 5 | 1 | 1.2 | 100 |
| **CB_06_v** | Victim | 0 | 379 | 73.7 | 91.5 | 91.5 |
|  |  | 1 | 35 | 6.8 | 8.5 | 100 |
| **CB_06_a** | Abuser | 0 | 413 | 80.4 | 99.8 | 99.8 |
|  |  | 1 | 1 | 0.2 | 0.2 | 100 |
| **CB_07_v** | Victim | 0 | 395 | 76.8 | 95.4 | 95.4 |
|  |  | 1 | 19 | 3.7 | 4.6 | 100 |
| **CB_07_a** | Abuser | 0 | 412 | 80.2 | 99.5 | 99.5 |
|  |  | 1 | 2 | 0.4 | 0.5 | 100 |
| **CB_08_v** | Victim | 0 | 406 | 79 | 98.1 | 98.1 |
|  |  | 1 | 8 | 1.6 | 1.9 | 100 |
| **CB_08_a** | Abuser | 0 | 410 | 79.8 | 99 | 99 |
|  |  | 1 | 4 | 0.8 | 1 | 100 |
| **CB_09_v** | Victim | 0 | 381 | 74.1 | 92 | 92 |
|  |  | 1 | 33 | 6.4 | 8 | 100 |
| **CB_09_a** | Abuser | 0 | 409 | 79.6 | 98.8 | 98.8 |
|  |  | 1 | 5 | 1 | 1.2 | 100 |
| **CB_10_v** | Victim | 0 | 410 | 79.8 | 99 | 99 |
|  |  | 1 | 4 | 0.8 | 1 | 100 |
| **CB_10_a** | Abuser | 0 | 410 | 79.8 | 99 | 99 |
|  |  | 1 | 4 | 0.8 | 1 | 100 |
| **CB_11_v** | Victim | 0 | 398 | 77.4 | 96.1 | 96.1 |
|  |  | 1 | 16 | 3.1 | 3.9 | 100 |
| **CB_11_a** | Abuser | 0 | 400 | 77.8 | 96.6 | 96.6 |
|  |  | 1 | 14 | 2.7 | 3.4 | 100 |
| **CB_12_v** | Victim | 0 | 383 | 74.5 | 92.5 | 92.5 |
|  |  | 1 | 31 | 6 | 7.5 | 100 |
| **CB_12_a** | Abuser | 0 | 391 | 76.1 | 94.4 | 94.4 |
|  |  | 1 | 23 | 4.5 | 5.6 | 100 |
