# Supplementary material for: Cybervictimization, self-esteem, and cyberaggression endorsement in Thai university students: evidence from a two-part zero-inflated model
Source: Front Psychol. 2026 Sep 11;17:1863842. doi: 10.3389/fpsyg.2026.1863842 (PMC13612487; doi:10.3389/fpsyg.2026.1863842)
Supplement: Supplementary file 3 [file Table_2.docx]

**Table S2. Correlations among study variables (N = 414)**

| **Variable** | **1** | **2** | **3** | **4** | **5** | **6** |
| --- | --- | --- | --- | --- | --- | --- |
| 1. Sex (male = 1, female = 0) | — |  |  |  |  |  |
| 2. Age | −.07 | — |  |  |  |  |
| 3. Internet use | .04 | −.21** | — |  |  |  |
| 4. Cybervictimization | −.05 | −.10* | .07 | — |  |  |
| 5. Cyberaggression endorsement (AGGRESSO) | −.09 | .05 | .03 | .33** | — |  |
| 6. Self-esteem | −.11* | .07 | −.06 | −.23** | −.14** | — |
