# Supplementary material for: Cybervictimization, self-esteem, and cyberaggression endorsement in Thai university students: evidence from a two-part zero-inflated model
Source: Front Psychol. 2026 Sep 11;17:1863842. doi: 10.3389/fpsyg.2026.1863842 (PMC13612487; doi:10.3389/fpsyg.2026.1863842)
Supplement: Supplementary file 4 [file Table_3.docx]

**Table S3. Covariate-Adjusted Sensitivity Analysis: Zero-Inflated Poisson (ZIP) Model Estimating Cyberaggression Perpetration (AGGRESSO)**

|  | Estimate | S.E. | Est./S.E. | P-Value |
| --- | --- | --- | --- | --- |
|  |  |  |  |  |
| AGGRESSO | ON |  |  |  |
| VICTIMC | 0.07 | 0.053 | 1.321 | 0.187 |
| ROSENC | -0.083 | 0.032 | -2.595 | 0.009 |
| INTXN_C | 0.018 | 0.012 | 1.58 | 0.114 |
| AGE | 0.045 | 0.194 | 0.231 | 0.817 |
| GENDER | 0.181 | 0.07 | 2.596 | 0.009 |
| USAGE | 0.16 | 0.197 | 0.811 | 0.418 |
|  |  |  |  |  |
| AGGRESSO#1 | ON |  |  |  |
| VICTIMC | -1.661 | 0.523 | -3.174 | 0.002 |
| ROSENC | -0.05 | 0.047 | -1.063 | 0.288 |
| INTXN_C | -0.122 | 0.044 | -2.764 | 0.006 |
| AGE | 1.012 | 0.443 | 2.283 | 0.022 |
| GENDER | 0.043 | 0.131 | 0.328 | 0.743 |
| USAGE | 0.069 | 0.349 | 0.198 | 0.843 |
|  |  |  |  |  |
| Means |  |  |  |  |
| VICTIM | 0 | 0.073 | 0 | 1 |
| ROSENBER | 0 | 0.247 | 0 | 1 |
|  |  |  |  |  |
| Intercepts |  |  |  |  |
| AGGRESSO#1 | -0.639 | 3.328 | -0.192 | 0.848 |
| AGGRESSO | -4.469 | 1.486 | -3.007 | 0.003 |
|  |  |  |  |  |
| Variances |  |  |  |  |
| VICTIM | 2.23 | 0.304 | 7.334 | 0 |
| ROSENBER | 25.249 | 1.928 | 13.093 | 0 |

CONFIDENCE INTERVALS OF MODEL RESULTS

|  | Lower0.5% | Lower2.5% | Lower5% | Estimate | Upper5% | Upper2.5% | Upper0.5% |
| --- | --- | --- | --- | --- | --- | --- | --- |
| AGGRESSO | ON |  |  |  |  |  |  |
| VICTIMC | -0.066 | -0.034 | -0.017 | 0.07 | 0.156 | 0.173 | 0.206 |
| ROSENC | -0.165 | -0.145 | -0.135 | -0.083 | -0.03 | -0.02 | -0.001 |
| INTXN_C | -0.011 | -0.004 | -0.001 | 0.018 | 0.037 | 0.041 | 0.048 |
| AGE | -0.455 | -0.335 | -0.274 | 0.045 | 0.364 | 0.425 | 0.544 |
| GENDER | 0.001 | 0.044 | 0.066 | 0.181 | 0.295 | 0.317 | 0.36 |
| USAGE | -0.347 | -0.226 | -0.164 | 0.16 | 0.483 | 0.545 | 0.667 |
|  |  |  |  |  |  |  |  |
| AGGRESSO | ON |  |  |  |  |  |  |
| VICTIMC | -3.01 | -2.687 | -2.522 | -1.661 | -0.8 | -0.635 | -0.313 |
| ROSENC | -0.172 | -0.143 | -0.128 | -0.05 | 0.027 | 0.042 | 0.071 |
| INTXN_C | -0.236 | -0.208 | -0.195 | -0.122 | -0.049 | -0.035 | -0.008 |
| AGE | -0.13 | 0.143 | 0.283 | 1.012 | 1.741 | 1.881 | 2.154 |
| GENDER | -0.295 | -0.215 | -0.173 | 0.043 | 0.259 | 0.301 | 0.382 |
| USAGE | -0.831 | -0.616 | -0.506 | 0.069 | 0.644 | 0.754 | 0.969 |
|  |  |  |  |  |  |  |  |
| Means |  |  |  |  |  |  |  |
| VICTIM | -0.189 | -0.144 | -0.121 | 0 | 0.121 | 0.144 | 0.189 |
| ROSENBER | -0.636 | -0.484 | -0.406 | 0 | 0.406 | 0.484 | 0.636 |
|  |  |  |  |  |  |  |  |
| Intercepts |  |  |  |  |  |  |  |
| AGGRESSO#1 | -9.211 | -7.162 | -6.113 | -0.639 | 4.835 | 5.884 | 7.933 |
| AGGRESSO | -8.297 | -7.382 | -6.914 | -4.469 | -2.024 | -1.556 | -0.641 |
|  |  |  |  |  |  |  |  |
| Variances |  |  |  |  |  |  |  |
| VICTIM | 1.447 | 1.634 | 1.73 | 2.23 | 2.73 | 2.826 | 3.013 |
| ROSENBER | 20.282 | 21.469 | 22.077 | 25.249 | 28.421 | 29.028 | 30.216 |

*Note. The model simultaneously estimates two components: (a) the* ***count model*** *(top panel, "AGGRESSO ON"), predicting the expected frequency of endorsed aggressive behaviors among individuals not in the excess-zero group; and (b) the* ***zero-inflation model*** *(bottom panel, "AGGRESSO#1 ON"), predicting the log-odds of being an excess-zero (i.e., consistently non-aggressive). Covariates include victimization (VICTIMC), self-esteem (ROSENC), interaction (INTXN_C), age, gender, and internet usage. Confidence intervals (CIs) are based on [specify, e.g., bootstrap percentile method with 5,000 resamples].*
